# Are demoralization and insight involved in suicide risk? An observational study on psychiatric inpatients

**DOI:** 10.1192/j.eurpsy.2021.462

**Published:** 2021-08-13

**Authors:** I. Berardelli, M. Innamorati, S. Sarubbi, E. Rogante, D. Erbuto, A. Costanza, A. Del Casale, M. Pasquini, D. Lester, M. Pompili

**Affiliations:** 1 Corresponding Author: Prof. Maurizio Pompili, Department Of Neurosciences, Mental Health And Sensory Organs, Suicide Prevention Center, Sant’andrea Hospital, Sapienza University Of Rome, 00189 Rome, Italy., Sapienza University of Rome, Rome, Italy; 2 Department Of Human Sciences, European University of Rome, Rome, Italy, Rome, Italy; 3 Department Of Psychology, Sapienza University of Rome, Rome, Italy; 4 Department Of Neurosciences, Mental Health, And Sensory Organs, Department of Neurosciences Mental Health and Sensory Organs, Faculty of Medicine and Psychology, Sapienza University of Rome, Rome, Italy; 5 Department Of Psychiatry, Faculty Of Medicine, University of Geneva (UNIGE), 1211 Geneva, Switzerland;, Ginevra, Switzerland; 6 Department Of Clinical And Dynamic Psychology, Sapienza University of Rome, Rome, Italy; 7 Department Of Human Neurosciences Faculty Of Medicine And Dentistry, Sapienza University of Rome, Rome, Italy; 8 Psychology Program, Stockton University, New Jersey, United States of America

**Keywords:** prevention, suicide risk, demoralization, insight

## Abstract

**Introduction:**

Although several authors have investigated the relationship between demoralization, insight, and suicide risk, the role of these factors in determining suicide risk in patients with psychiatric disorders is still unclear [Berardelli et al., 2019; Costanza et al., 2020].

**Objectives:**

The main aim of this study was therefore to determine whether suicide risk was associated with better insight and worse demoralization in a sample of 100 adult psychiatric inpatients.

**Methods:**

The study was performed on 100 psychiatric hospitalized adult patients consecutively enrolled between January 2019 and April 2020 at psychiatric units of Sant’Andrea Medical Center, Sapienza University of Rome. The Columbia Suicide Severity Rating Scale (C-SSRS) was used to assess suicide risk, Demoralization was assessed using the Demoralization Scale (DS) [Kissane et al., 2004] and for the assessment of insight we used the The Insight Scale (IS).

**Results:**

Only age was significantly associated with higher suicide risk (χ2=9.07, p<0.01). The variable mood disorder was significantly associated with higher suicide risk (χ22=7.50, p<0.05). Non-suicidal self-harm behaviors in the last 3 months (χ2=5.89, p<0.05) and lifetime suicide attempts (χ2=21.80, p<0.001) were significantly associated with higher suicide risk. Only the insight-high dimension (χ2=8.01, p<0.01) and lifetime suicide attempts (χ2=12.33, p<0.001) were significantly associated with higher suicide risk.

**Conclusions:**

Our results don’t confirm the role of demoralization in suicide risk. In our sample of patients, only high insigth of illness and other psychological variables are involved in suicide risk.

**Disclosure:**

No significant relationships.

